# Practical Observations on Some Diseases of the Stomach and Alimentary Canal

**Published:** 1847-04-01

**Authors:** 


Practical Observations on some of the Diseases of the Stomach and Alimentary Canal. By *James Alderson,* M.D. F.R.S. late Senior Physician of the Hull General Infirmary, &c. &c. 8vo. pp. 215. With Ten Coloured Plates. Longman and Co. London, 1847.

This volume contains part of the gleanings, in one particular field of professional research, of many years' extensive and carefully-observed experience in one of our large provincial towns and its neighbourhood. The work is thoroughly practical, and is but the faithful record of what the author has himself witnessed by the bed-side of the sick. He has wisely avoided enlarging its dimensions by the too common practice of introducing large quotations from the writings of others. There is a two-fold division of its contents; the first part being occupied with the consideration of some of the Structural Diseases of the Stomach and Intestines, and the second with that of some of their Functional Disorders. We shall be able to notice the former only.

Dr. Alderson does not enter upon the histological characters of Carcinomatous deposits; nor is it necessary for us to allude to them again, after the ample exposition which we gave, in a late Number of this Journal, of all the most recent discoveries on the subject in our review of Dr. Walshe's classic work.*

Next to the Uterus, the Stomach is the viscus of the body most frequently affected with Carcinoma. All its three forms—the scirrhous, the encephaloid, and the colloid or gelatinous—are met with in this organ; indeed, it is the special seat of the last-named form or species. The morbid deposit very generally occurs as an infiltration in the submucous cellular tissue. The pylorus is the part of the stomach most frequently affected; then the cardiac orifice; next the great, and lastly the lesser, curvature. More than two-thirds or even three-fourths of the organ may be involved in the destructive disorganising process. When the disease is so extensive, it is generally of the colloid species. The muscular coat of a stomach affected with Carcinoma is usually thickened and hypertrophied; occasionally however—but this is rare—it becomes attenuated and wasted. The size of the organ varies much in different cases. It has been known to be so much expanded, that it nearly reached to the pubes; in other cases it has been found greatly shrunk and contracted. The former condition is present, when the pyloric orifice has become narrowed from the disease; while the latter is generally associated with a like condition of the cardiac.

Carcinoma of the Stomach is more frequent in the male than in the female sex. It seldom occurs before 35 or 40 years of age. In the following extract, it will be observed that our author associates together the Encephaloid and Colloid species of the disease under the common appellation of "areolar."

"Of the various forms under which carcinoma attacks the stomach, there are two which, though usually considered as varieties of the same disease, tend to such different results when situated at either orifice, that I am induced to consider them separately. I am willing to admit that they have a common origin, and are only modified in their progress by incidental circumstances.

"One of these forms tends to close the orifice or outlet by the deposit of a dense cartilaginous-like material, constituting scirrhous stricture (Simple Carcinoma). This form (scirrhous stricture) has very little, if any, of a softer material in its composition, and, when it is ulcerated, the abraded surface is small, and the ulceration superficial. The effect of the deposit is occlusion of the orifice, whether of the cardia or of the pylorus.

"The other form, however the deposit may have first commenced in the part, is found, after death, in a softened, ulcerated state, and the outlet is comparatively

---

* We have much pleasure in referring our readers to some valuable observations on Cancer in the last Number (for March) of the Edinburgh Journal of Medical Science, by Dr. J. Hughes Bennett. This distinguished observer discusses in his paper the following four questions connected with the disease, which more particularly interest the medical practitioner; viz.—1. Is there any anatomical character which will enable us positively to distinguish a cancerous from any other kind of growth? 2. Is there any evidence that Cancer is ever spontaneously curable? 3. What means do we possess of diagnosing cancerous from other tumours or growths in the living subject? and 4. What influence should our present knowledge of diagnosis have upon the treatment?

open. The deposit is of a mixed character, varying in density from that of scirrhus to the softness of brain or pulp: and the ulcerated surface is always extensive, and covered with fungous projections. This form is called Areolar Carcinoma.

" However true it may be that these varieties are originally identical, and only in their subsequent development modified by incidental circumstances, I think it better to consider them separately, and endeavour, by a comparison of the symptoms during life with the appearances after death, to accumulate signs by which we may be able to distinguish them from each other in the living subject. Both varieties are alike constitutional, and both, when developed in certain situations in the stomach, are attended by the secondary deposit of encephaloid or medullary matter in the other viscera, especially in the liver: the particular situations will be hereafter indicated. The secondary deposit in the liver consists always of a soft homogeneous matter dispersed throughout the organ in the form of tubera, which, from their consistence, have been called encephaloid or medullary.

" In consequence of the mechanical functions performed by the orifices of the stomach, which, when in a natural state, and not in action, are always contracted, we find the coats at the orifices to be composed of a denser and more compact structure than the rest of the stomach; and it may be to this peculiarity of structure that we must attribute in them the prevalence of the scirrhous form of carcinoma, uncomplicated, or nearly so, with areolar deposit, a supposition borne out by the fact that we never meet with scirrhus in its simple form in the larger curvature of the stomach." P. 15.

One of the most prominent symptoms of *Carcinoma* (whether in the scirrhous or areolar form) *of the Cardiac orifice* is the rejection of the food immediately, or very soon, after it has been swallowed.

" The common saying among hospital patients is, 'that one mouthful brings another up,' which mode of expression, though rude, is almost literally true, since the distension of the œsophagus by a second mouthful causes the whole to be immediately rejected. This is sometimes accompanied by a sense of suffocation: the patient feels the food go to a certain spot, and then stop: pain is immediately experienced at that point, and vomiting succeeds. The pain is of a dull, aching kind, and strikes through to the back and between the shoulders.

" In the earlier stage and progress of the disease, vomiting does not occur invariably after taking food, but it is never absent during many days together; the interval gradually diminishes as the disease advances: when stricture takes place, vomiting becomes a constant symptom, always following immediately when nourishment has been taken. The food then returns in the same state as it has been swallowed, or mixed with ropy mucus, secreted by the lining membrane of the œsophagus. Ultimately, even liquids will not pass into the stomach, and the patient having subsisted upon his own fat, at last dies, emaciated, of starvation." P. 21.

The amount of suffering from Carcinoma of the Cardia is, as might be expected, greatly more severe, but much less protracted than when the Pylorus is the seat of the disease. In some cases, not a mouthful of food can find its way into the stomach, while the sharpest hunger is experienced all the while. There is then no other way of supporting life, except by the use of nutritious enemata.

In no instance of cardiac Carcinoma, occurring in the practice of our author, did he discover on dissection any traces of encephaloid deposit in the liver.

When *Carcinoma* occurs *in the larger Curvature of the Stomach,* it is invariably of the areolar form; true scirrhus being never met with in this part. Its presence is

" Attended by symptoms of dyspepsia in an aggravated form, by vomiting at variable periods after taking food, by the escape of fetid air from the stomach, and usually by great pain at the pit of the stomach, which is increased by the slightest pressure.

" There is a sense of great debility. The countenance is very anxious, and the spirits depressed; the complexion is exsanguineous and opaque; the tongue is always clean; the state of the bowels varies in different cases, but is usually confined.

" In some few cases vomiting does not occur, and frequently, in the later stages of the complaint, it is superseded by 'sour risings,' by fetid 'mouthfuls,' which are brought up from the stomach.

" Less pain is experienced in this situation of the disease than when it occurs at either of the outlets. Emaciation takes place, but not to the extreme extent which accompanies scirrhous cardia.

" There is always deposit of encephaloid tubera in the liver." P. 53.

As long as the Cardiac and Pyloric orifices remain sound, food can be received into the stomach; but the organ never contracts with its normal force upon its contents, and thus a greater or less degree of difficulty is always experienced in propelling the half-digested mass, either upwards into the œsophagus, or downwards into the intestinal canal. On examination by the touch, a tumour may very generally be detected in the epigastric region; as the disease, however, advances, this becomes increased in size, but less circumscribed and definite in form.

" The increase of the mass, in this case, is to be attributed to the encroachment of an enlarged liver, and to subsequent adhesions between the liver, the stomach, and neighbouring organs. In consequence of the increase, the position of the tumour is no longer to be distinctly referred to one spot; and that, which was previously a comparatively distinct tumour, is superseded by a gradually increasing mass, which extends across the pit of the stomach, and below the cartilages of the ribs." P. 54.

*Carcinoma of the Pylorus.*—The pyloric extremity of the stomach, as we have already said, is by far the most frequent seat of malignant degeneration (which is generally of the areolar kind); and there is less difficulty in forming a correct diagnosis as to the disease, when it is situated in this than in any other part of the viscus. The tumour in the epigastrium is usually distinct and well defined, until adhesions between the stomach and the adjacent viscera have taken place, and then its form and outline become less distinguishable. The commencement and progress of the disease are usually indicated in the following manner:

" Pyloric disease at its onset is usually marked by symptoms of dyspepsia. They are urgent and distressing, and do not yield to the remedies which are found to avail in functional disorders of the stomach. These will have been present for about two years before the disease approaches to a crisis. In the latter year the change to the true cancerous complexion marks the uncontrolled advance of structural disease. With a few exceptions, pain, which is aggravated by touch or pressure, is experienced in the region of the stomach, to the right of the ensiform cartilage. During the examination, when the diseased part is touched, it is usual for the patient, though suffering severely from the ordeal, to express a peculiar and melancholy satisfaction that you have touched the precise point from which all his pain proceeds.

" Sickness is also generally a marked symptom of this situation of the disease; it comes on two or three hours after taking food, and is almost always preceded by pain." P. 76.

In some cases, indeed, little pain or vomiting is experienced; but the absence of these symptoms is, as might be supposed, quite an exceptional occurrence. The sickness is sometimes suspended for a period of several days at a time.

" I have known an interval of fourteen days between the vomiting, when all at once the accumulation of nearly all that had been taken during the interval was vomited in a chymified state, mixed with mucus, or with vitiated secretion from the ulcerated surfacesd. The greatest relief is experienced after this complete emptying of the stomach; and when it occurs for the first time, the patient has a delusive conviction that the complaint is finally subdued. The accumulation, however, soon again takes place; and it is painful to witness the corresponding depression which succeeds this temporary but disappointed hope. It is usual at this time for the spirits to begin to droop, and the patient is unable, upon the faintest excitement, to restrain emotion, even to tears." P. 78.

Ulcerated Carcinoma of the Pylorus is almost invariably associated with Encephaloid disease in the Liver; and not unfrequently, when the nodules or tubera of this morbid deposit are situated on the anterior surface of this viscus, they may be detected by careful examination through the abdominal parietes. Jaundice is not an uncommon result of this hepatic complication. The gall-bladder is almost always found to contain gall-stones. Dr. A. remarks that, several of his patients, who were the subjects of cancer of the pylorus, had previously suffered from attacks of gall-stones.

*Carcinoma External to the Stomach.*—The following narrative of the pathological changes alluded to under this appellation will best enable the reader to appreciate the value of Dr. Alderson's descriptions.

"Deposit of carcinomatous matter may take place beneath, or on the free surface, of the serous membrane covering the stomach, and the neighbouring viscera (as the duodenum, the head of the pancreas, the omenta, the mesocolon, &c.): the deposit varies in consistence from the hardness of scirrhus to the softness of pulp. It is difficult, if not impossible, to assign a position to the earlier deposit; as it increases, however, lymph is effused, and adhesions are formed, by which contiguous parts are glued together; the viscera become bound to the spine, and to each other, by these adhesions, and, as a consequence, the healthy performance of their various functions is interfered with. The adhesions often present a dark smoky hue, and jelly-like appearance, and are easily torn through: the proximity of organs, rather than the similarity of function, seems to be the cause of their being involved. The order in which the parts are severally included in the diseased mass may be inferred from the succession of the symptoms which will be hereafter detailed: this order, as well as the number of organs finally included, varies in different cases. In some cases the disease proceeds until portions of the stomach, the duodenum, the pancreas, the colon, and even the liver, are all involved in one diseased mass, forming an undefined tumour. In the later stages of the disease ulceration finds its way into the duodenum, the stomach or the colon. The ulceration has a peculiar character, differing from that which results when the primary seat of the deposit is in the submucous cellular tissue: the disorganising process is, perhaps, more properly expressed by erosion, which takes place through the coats, where either hard or soft material of carcinoma is deposited." P. 98.

The Symptomatology of the disease is thus very accurately given:

"Amongst the earlier symptoms of this disease, unsatisfactory relief from the bowels is the most prominent. It is experienced for a year or more before the more urgent symptoms set in, and is accompanied at intervals by nausea, retchings, and headache. Pain is also suffered, but not to any great extent, and it is referred by the patient to an unremoved accumulation in the bowels; it is described as situated at the pit of the stomach, or rather lower. The patient subsequently begins to lose flesh, the features shrink, the complexion becomes opaque, sallow, and exsanguineous; the eye looks sunken, and the strength begins to fail; great anxiety about his health now possesses the patient, who is often prone to seek various opinions, until he finds a voice ready to flatter him with the delusive promise of a cure.

"At this early period the tongue is tolerably clean, but not in the peculiar degree met with in carcinoma of the interior of the stomach; the pulse is, on the whole, natural, but inclined to be weak and frequent; there is often morbid appetite, and considerable thirst; sometimes a degree of voracity for food, whether solid or liquid.

"The accumulation in the colon really exists, and is with difficulty dislodged. Pyrosis often supervenes, with the peculiar drawing-in of the diaphragm to the spine.

"Within three or four months of a fatal termination a careful examination of the abdomen usually reveals an unusual fulness a little to the right of the pit of the stomach; it is hardly felt as a tumour, but as an undefined resisting mass, and it appears to rest upon the spine: pressure by the hand causes pain; and, the same as in cases of ulcerated carcinoma of the stomach, I have several times noticed that the patient exclaims with satisfaction that the precise seat of the pain has been touched, and he generally adds his conviction that the cause will be removed.

"At this time loss of flesh and strength increases, the appetite fails entirely, there are vomitings and retchings: the nights are restless, attended with pain. Hiccup is a very distressing symptom, and increases both in violence and in duration of the fit as the disease advances. The tongue shows a tendency to aphthæ, which also gradually increase. Local irritation on the skin, as erythema and erysipelas in the lower extremities, sometimes appear; and whilst they are present there appears some little remission of the other symptoms. Diarrhœa of severe character sets in at intervals: jaundice, with all the symptoms attendant upon it, often supervenes about this stage of the disease." P. 102.

In the closing scene, the prostration is sometimes extreme; the patient is exhausted with hectic fever; and vomiting of dark-coloured fluid, or even of blood, is generally present in a greater or less degree.

It is a melancholy feature of the disease which we have been considering, that in its early stage it is generally mistaken for mere functional derangement of the stomach, and treated accordingly;—by remedies too that often serve but to aggravate the existing lesion. We have already seen that one of the most constant symptoms, at the commencement of the disease, is the sensation of load in the bowels; a sensation which is somewhat relieved indeed by free alvine discharges, but only to return with as much distress as ever. There is, in many cases, a large accumulation of fæculent contents in the colon during the course of the malady—(a circumstance which may lead a physician to suspect a much wider extent of morbid deposit than really exists); but, even when these have been duly evacuated, the uneasiness in question is found to be scarcely lessened. In our author's opinion, it "is rather to be attributed to the presence of carcinomatous deposit, and its progressing adhesions, than to accumulation within the bowel, to which the patient always attributes it." We should, therefore, be on our guard not to have recourse to unnecessarily active medicines, in the vain hope of giving any material relief to symptoms which are inevitable. The use of any but the very gentlest aperients is still the more necessary, when there is reason to believe that the morbid process has involved any portion of the gastro-intestinal mucous surface.

This remark naturally leads us to the subject of the *treatment* of Carcinoma of the Stomach. The skill of the kind and wise physician consists rather in guarding his patient from injudicious, and perhaps hurtful, medication than in seeking to arrest, or even to materially mitigate, the fearful disease that he has to contend with. Our author writes with no less good feeling than sound judgment in reference to the sad duty which a medical man is called upon in such a case to fulfil, when he seeks to comfort where he dare not promise, to calm and tranquillize where he cannot encourage. His principal remarks on the therapeutic part of his subject is contained in this passage:

"As soon as the symptoms fairly indicate that the disease is in the larger curvature, or in the pylorus, as we have endeavoured to show they may do before ulceration commences, and long before positive evidence is given in the form of tumour, a course of active counter-irritants should be immediately adopted. Local depletion, either at the pit of the stomach or over the spine, may succeed in checking or retarding the irritation of the mucous coat, and blisters may be applied as near to the seat of pain as possible. It is necessary at the same time to discontinue all aperient medicines which tend to irritate the mucous membrane of the stomach, and to choose such as act upon the colon, since we must seek to obtain enlarged secretion from the mucous membrane of the bowels, both as a counter-irritant and as a measure of depletion. The occasional and moderate use of calomel, unmixed with any irritating purgative, or of hydrargyrum-cum-creta, is a most useful adjunct. There is evidence in several ways that counter-irritation has apparent power in this disease. The appearance of gout and erysipelas seem to stay the progress for a time. In Case No. 8, there is an instance of an external ulcerated tumour in the groin, which seemed to divert the course of the disease.

" Simple astringents, as lime-water given in milk, and sedatives, may be usefully employed in this stage of structural disease, in the same way as if the secretions, intended to be corrected, were the result of mere disordered function. All stronger astringents should be carefully avoided. The opiates should be sparingly had recourse to, as there will be too much occasion for their aid in succeeding periods of severer trial; they may be usefully combined with alkalis, if indicated, and with hydro-cyanic acid. The diet should at once be changed, to avoid all irritating, stimulating food. The simple animal food which is so useful in common dyspepsia will be too irritating for this stage; beef and chicken tea would not be objectionable, but there is generally a distaste for them. Milk is the only animal food which is generally agreeable, and it is highly appropriate. Pure air, quiet, rest from great exertion, whether mental or bodily, but especially the latter, should be sought." P. 150.

The alterative most worthy, in his opinion, of a fair trial in the early stage of cancerous development is the Bichloride of Mercury, in minute doses conjoined with Sarsaparilla. The extract of Conium has been found one of the safest and most useful anodynes. The application of a Belladonna plaster to the epigastric region at the same time may assist its soothing action.

Dr. Alderson has a chapter on *Hypertrophy of the Coats of the Stomach.* The disease is limited to the pyloric extremity of the organ, and is almost always associated with—perhaps generally dependent upon—a contracted state of the pyloric orifice. It appears to be generally attributable to chronic inflammation of the mucous coat in the first instance, this inflammation extending to the other coats of the stomach in consequence of the use of stimulating food and drinks.

" This disease is preceded by morning sickness, and want of appetite; in the later stages the general symptoms are constant retching and sickness; great impatience for drink, which, of whatever quality, and in whatever quantity, is always immediately rejected. Dark-coloured fluid is also vomited in large quantity. Everything taken into the stomach gives pain, and causes not only its own rejection, but also copious vomiting of vitiated secretion from the mucous lining of the stomach. There are heats and chills, and the pulse is quick and the tongue furred. Pain is usually felt on pressure at the pit of the stomach, but not invariably; more so, probably, in cases which owe their origin to unsubdued chronic inflammation than to mere obstruction at the pylorus.

" Emaciation is not a symptom of the disease, and hence its absence negatively distinguishes the complaint from carcinoma." P. 132.

Two interesting cases are related. In both, the prominent and most distressing symptom was an almost incessant vomiting. We are tempted to give the report of the second case complete.

" Mr. H., aged 49, a traveller for a mercantile house in the wine and spirit trade. He had been an active man of business, and in the course of seeking orders for his trade, had been led into convivial habits, which increased to an extreme degree. When I first saw him he was vomiting continually, always calling for liquids, toast and water, soda water, and even brandy and water; all of which were immediately rejected, together with a large quantity of mucous secretion, from the stomach. He was moaning, and restless, and seemed to find most relief by lying upon his stomach, rather on one side, and was very disinclined to be questioned, though anxious for relief. His pulse was small and quick, his tongue furred, and the urine high-coloured.

" On examination, the slightest pressure at the pit of the stomach caused him great pain. He said, *there* was situated all his misery, and unless something were done, and soon, to relieve it, he must die.

" All these symptoms had come on about a week before, and had continued increasing to the period of my seeing him. For a long time previous to their appearance he had suffered from morning sickness, want of appetite, occasional pain, and flatulence. He attributed his discomforts to his mode of life, and confessed that he usually sought to relieve them by additional stimulants. He was still a fine-looking, muscular man, not at all emaciated.

" General depletion was not ventured upon, as he had recently been the subject of delirium tremens; the most active local antiphlogistic measures were resorted to, but without giving relief. Nothing which was done seemed to have any control over the sickness, which was continued to the last. He only lived a few days.

" *Examination of the body* 24 *hours after death.*—On opening the body the parieties were well stored with fat.

" The stomach was the seat of considerable change; the lining membrane was of a dull red colour; the opening into the duodenum was narrowed, but freely admitted the finger; the muscular and mucous coats were found hypertrophied. The muscular coat was firm, rather than hard like scirrhus; the mucous coat softer than natural, and thickened and injected." P. 138.

We must now take leave of Dr. Alderson. The perusal of his present volume has afforded us much gratification, and we trust that ere long we shall again have the pleasure of introducing him to the notice of our readers.

